# P-1047. Incidence and Characteristics of Ventilator-Associated Events (VAEs) in an adult ICU

**DOI:** 10.1093/ofid/ofaf695.1242

**Published:** 2026-01-11

**Authors:** Sunit Sikdar, Seema Sood, Manish Soneja, Animesh Ray, Megha Priyadarshi, Bimal Kumar Das, Naveet Wig

**Affiliations:** All India Institute of Medical Sciences, New Delhi, Delhi, Delhi, India; All India institute of medical sciences, new delhi, NEW DELHI, Delhi, India; All India Institute of Medical Sciences, New Delhi, Delhi, India; All India Institute of Medical Sciences, New Delhi, Delhi, Delhi, India; All india institute of medical sciences, Delhi, Delhi, India; All India Institute of Medical Sciences, New Delhi, Delhi, Delhi, India; All India Institute of Medical Sciences, New Delhi, Delhi, India

## Abstract

**Background:**

Ventilator-Associated Events (VAEs) were introduced by the Centers for Disease Control and Prevention (CDC) to replace traditional ventilator-associated pneumonia (VAP) definitions. This shift aims to reduce subjective variability, improve the reproducibility of surveillance, and capture both infectious and non-infectious complications in mechanically ventilated patients.

**Figure:**
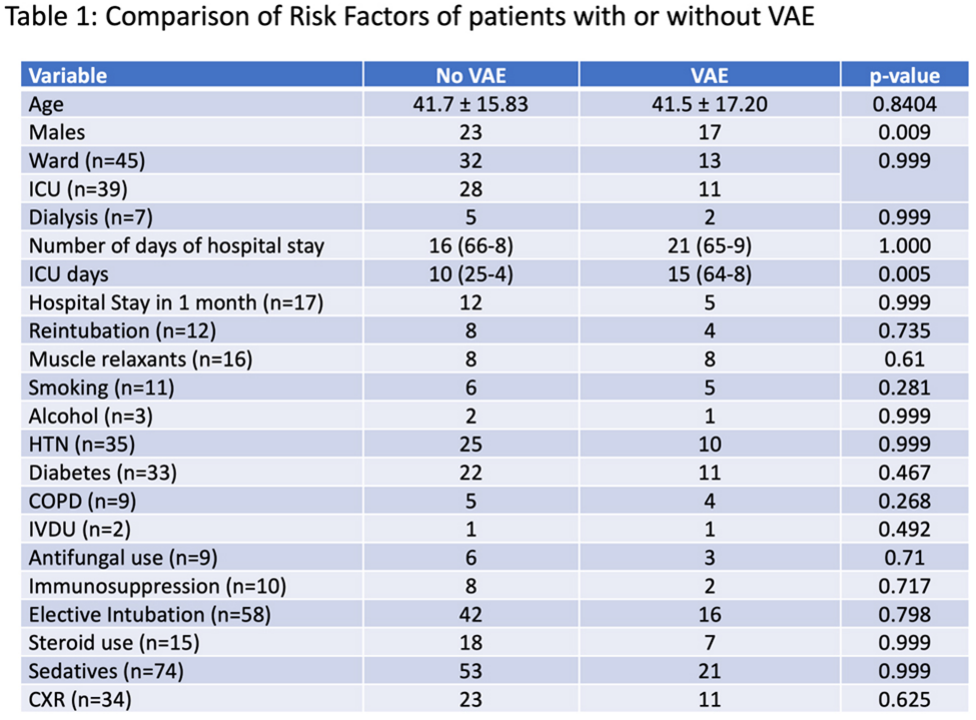

**Figure:**
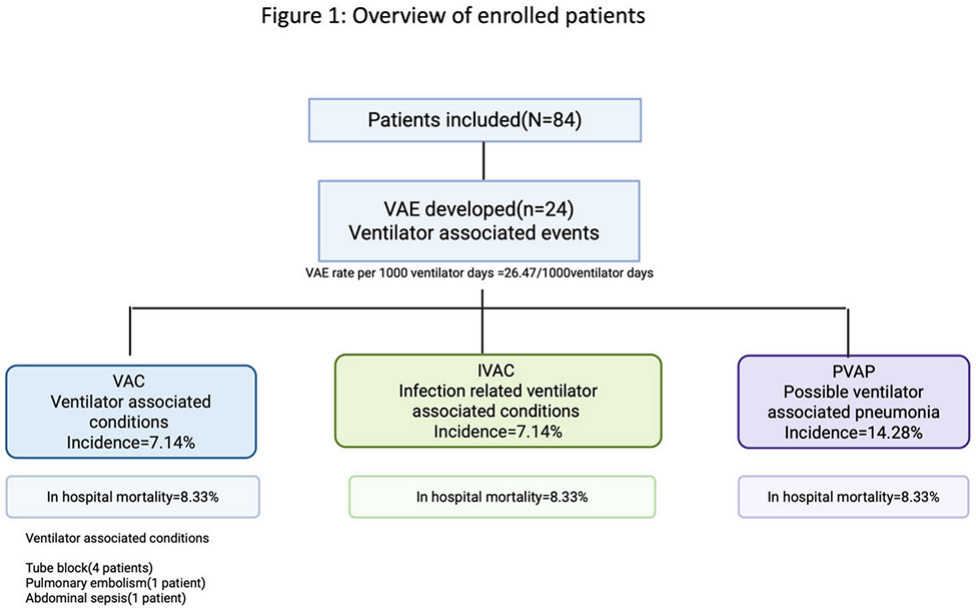

**Methods:**

A prospective observational study was conducted in an adult ICU at a tertiary care center from January 2023 to December 2024. Patients aged ≥18 years, on invasive mechanical ventilation for at least 48 hours, were enrolled. Demographic, clinical, and laboratory parameters were recorded. VAEs were classified according to CDC criteria into Ventilator-Associated Condition (VAC), Infection-related VAC (IVAC), and Possible Ventilator-Associated Pneumonia (PVAP). Respiratory samples (endotracheal aspirates, bronchoalveolar lavage, and protected specimen brush) were collected, cultured, and analyzed for pathogen profiles. Outcomes—including mortality, length of hospital and ICU stay—were assessed using appropriate statistical methods.

**Figure:**
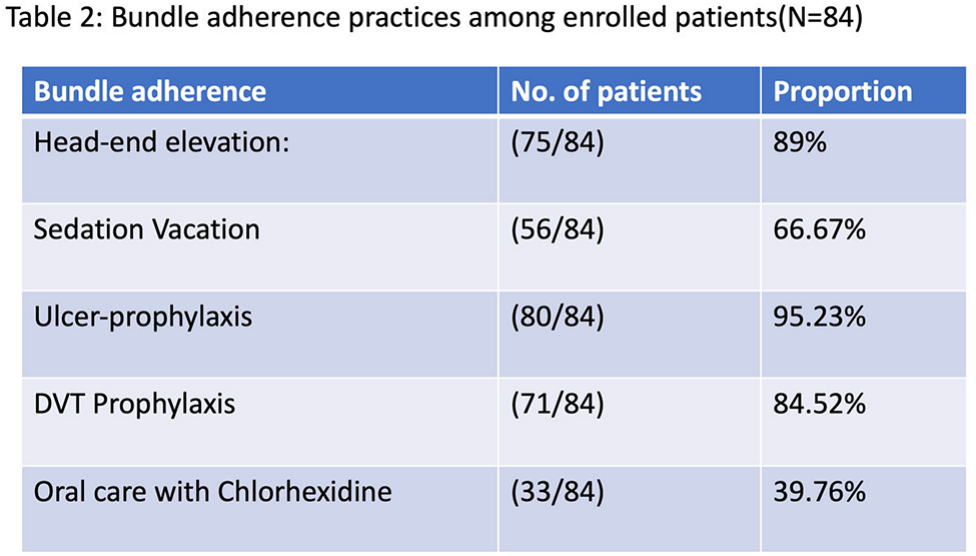

**Figure:**
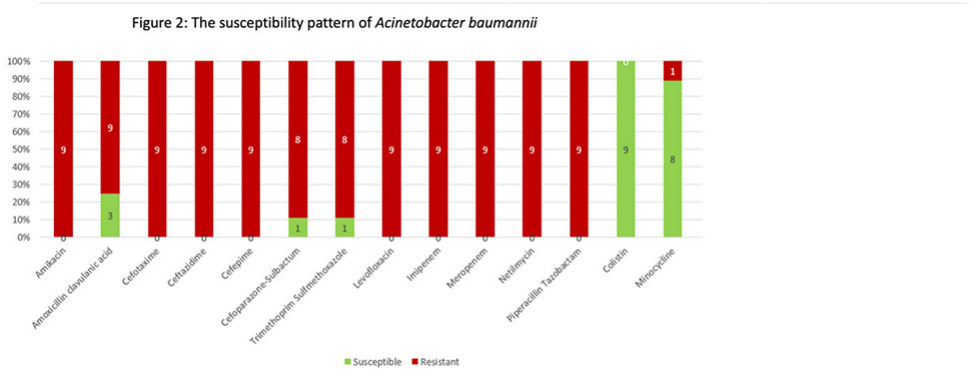

**Results:**

Among 84 enrolled patients (mean age 41.9 years), the overall VAE incidence was 28.57%, translating to 28.8 per 1000 ventilator-days. Sub-classification revealed rates of 7.2 per 1000 ventilator-days each for VAC and IVAC, and 14.44 per 1000 ventilator-days for PVAP. The predominant organism in PVAP was carbapenem-resistant *Acinetobacter baumannii*

, susceptible primarily to colistin. Male gender and prolonged ICU stay were significantly associated with VAE development. Adherence to ventilator bundles did not significantly reduce VAE incidence. Overall mortality among VAE patients was 15%.

**Conclusion:**

Standardized VAE criteria facilitate the timely detection of respiratory deterioration in ventilated patients. Enhanced infection control measures, personalized care, and targeted surveillance are paramount to reducing VAE rates and improving patient outcomes.

**Disclosures:**

All Authors: No reported disclosures

